# Transperineal ultrasound–based outcomes of postpartum pelvic floor recovery after elective cesarean section versus vaginal delivery: a systematic review and meta-analysis

**DOI:** 10.3389/fgwh.2026.1808222

**Published:** 2026-08-12

**Authors:** Chunhua Zhan, Nina Tu, Haoyuan Huang, Xueli Lai

**Affiliations:** 1 Department of Ultrasound Medicine, The Third Affiliated Hospital of Gannan Medical University, Ganzhou, Jiangxi, China; 2 Physical Examination Department, The Third Affiliated Hospital of Gannan Medical University, Ganzhou, Jiangxi, China; 3 Physiatry Department, The Third Affiliated Hospital of Gannan Medical University, Ganzhou, Jiangxi, China

**Keywords:** **Systematic Review Registration**: identifier CRD42024588537., elective cesarean section delivery, meta-analysis, pelvic floor recovery, pelvic organ prolapse, stress urinary incontinence, transperineal ultrasound, vaginal delivery

## Abstract

**Objective:**

To synthesize evidence on transperineal ultrasound-based and clinically relevant postpartum pelvic floor outcomes following elective cesarean section delivery (CSD) compared with vaginal delivery (VD).

**Methods:**

Observational studies (cohort, case-control, and cross-sectional studies) comparing elective CSD with VD were included. Outcomes were pelvic floor muscle strength (PFMS), posterior bladder angle (PBA), bladder neck descent (BND), postpartum SUI, and POP. Risk of bias was assessed using ROBINS-I, and certainty of evidence was evaluated using the GRADE framework.

**Results:**

Twelve studies contributed to at least one outcome-specific quantitative or narrative synthesis. Five studies were included in the PFMS analysis. Because the included studies used different PFMS measurement methods and scales, PFMS was analyzed using standardized mean difference (SMD) rather than mean difference. Reported elective CSD showed a non-significant trend toward higher PFMS compared with VD (SMD = 0.27, 95% CI −0.05 to 0.60, *p* = 0.10, *I*^2^ = 74.75%). PBA did not differ significantly between delivery modes (3 studies, MD = −3.26, 95% CI −7.09 to 0.56, *p* = 0.09, *I*^2^ = 71.85%). BND was reported in only two studies and was summarized narratively. Reported elective CSD was associated with a lower risk of postpartum SUI than VD (6 studies, RR = 0.47, 95% CI 0.38 to 0.59, *p* < 0.001, *I*^2^ = 46.6%). The POP estimate also favored reported elective CSD (4 studies, RR = 0.56, 95% CI 0.35 to 0.91, *p* = 0.018, *I*^2^ = 74.0%), but was less robust because it was based on four studies with substantial heterogeneity. Labor exposure before cesarean delivery was incompletely reported in most studies, and postpartum assessment timing varied across studies.

**Conclusions:**

Current observational evidence suggests that reported elective CSD may be associated with a lower risk of postpartum SUI compared with VD. The evidence for POP also favored reported elective CSD, but was less robust. Evidence for PFMS and ultrasound-derived anatomical parameters remains uncertain. These findings should not be used as the sole basis for delivery-mode counseling. GRADE certainty was low for the SUI association and very low for PFMS, PBA, BND, and POP.

## Background

Postpartum pelvic floor dysfunction (PFD) is a common and clinically consequential condition that can impair women's long-term health and quality of life through symptoms such as stress urinary incontinence (SUI), pelvic organ prolapse (POP), pelvic floor pain, and reduced pelvic floor muscle performance. Current urogynecology emphasizes earlier identification of risk and personalized management across the perinatal period, because untreated dysfunction may persist and progress, creating substantial individual and healthcare burden (1, 2). Epidemiologic work in postpartum populations similarly indicates that PFD is frequent and multifactorial, supporting the need for objective assessment tools and prevention-oriented care pathways (3, 4).

Delivery mode has been discussed as one potentially relevant factor in postpartum pelvic floor recovery. During vaginal delivery (VD), passage through the birth canal may place substantial mechanical stress on the pelvic floor muscles, ligaments, and connective tissue, thereby increasing the risk of structural injury and altered pelvic support. Trauma to the levator ani muscle complex has been associated with pelvic floor morbidity, including prolapse-related and defaecatory dysfunction, supporting a plausible anatomical pathway linking birth exposures with later symptoms (5, 6). Cesarean section delivery (CSD), particularly when performed electively, avoids the second stage of labor and direct stretching of the levator hiatus and periurethral support. However, the degree and persistence of any lower-risk association remain debated, because pregnancy itself can induce hormonal and biomechanical remodeling that may contribute to pelvic floor dysfunction regardless of delivery mode (1).

Transperineal ultrasound has become an important modality for postpartum pelvic floor assessment because it is noninvasive, feasible, and able to quantify dynamic pelvic floor behavior at rest, during Valsalva, and during contraction. Ultrasound-derived measurements provide objective anatomical and functional parameters that complement symptoms and physical findings, including bladder neck support and pelvic floor geometry, which may be relevant to the mechanisms of SUI and POP (7, 8). Methodological advances, including automated or texture-based ultrasound analysis, may further improve reproducibility and support the use of ultrasound for postpartum surveillance and follow-up (9). Early postpartum imaging has also been explored as a tool for predicting later anorectal dysfunction and other pelvic floor sequelae, supporting the hypothesis that imaging markers could help identify women who may benefit from earlier intervention (10, 11).

Despite the increasing use of perineal ultrasound, the evidence comparing elective CSD with VD remains heterogeneous. Studies differ in design, postpartum assessment windows, ultrasound protocols, and outcome definitions, which complicates interpretation and quantitative synthesis (7). Intrapartum variables, particularly labor induction and labor exposure, may also affect pelvic floor morphology and confound delivery-mode comparisons (12). Maternal characteristics, including age, parity, BMI, and baseline pelvic floor risk, may further shape the recovery trajectory (4, 13). These sources of variability highlight the need for a structured synthesis focusing on both objective imaging outcomes and clinically relevant endpoints.

Ultrasound may also support postpartum prevention and rehabilitation strategies. Structured pelvic floor muscle training (PFMT) has shown benefits when evaluated using ultrasound and clinical outcomes, suggesting that imaging can serve as an outcome tool for monitoring recovery and response to rehabilitation (14). Beyond rehabilitation monitoring, prediction models that combine ultrasound markers with clinical measures have been proposed for identifying women at elevated risk of postpartum SUI and broader PFD, indicating a clinically meaningful direction toward risk-stratified follow-up (15, 16). However, before imaging-based screening or targeted pathways can be broadly recommended, clinicians require clearer quantitative estimates of how elective CSD and VD differ across key postpartum pelvic floor recovery outcomes.

Therefore, a systematic review and meta-analysis synthesizing perineal ultrasound–based pelvic floor recovery outcomes after elective cesarean section vs. VD is timely. By pooling evidence across available studies and focusing on comparable ultrasound endpoints and symptom-related outcomes, this approach can strengthen the evidence base for postpartum counseling, delivery-mode discussions, and risk-informed follow-up (1). No included study directly compared rehabilitation programs; therefore, rehabilitation planning was not an outcome of this review.

## Methods

### Study design and reporting

This study was conducted as a systematic review and meta-analysis comparing postpartum pelvic floor recovery outcomes after elective CSD vs. VD, with outcomes assessed using transperineal/perineal ultrasound and related clinical endpoints. The review was prepared in accordance with the Preferred Reporting Items for Systematic Reviews and Meta-Analyses (PRISMA) statement. This study was registered in PROSPERO under the registration CRD 42024588537. Statistical analyses were performed using Stata (version 18.0).

### Eligibility criteria

Studies were eligible for inclusion if they met the following criteria: (1) Participants comprised postpartum women who had delivered via elective cesarean section delivery (CSD) or VD. Elective CSD was defined according to the definition used in each original study. Studies were retained only when the cesarean group was described as elective CSD or when elective CSD data were clearly separable from emergency or mixed cesarean delivery. Where the original report did not explicitly state whether labor exposure occurred before cesarean delivery, this information was recorded as not reported or unclear. (2) Exposure and comparator were defined as elective CSD (exposure) compared with VD (comparator). (3) Outcomes included at least one of the following: pelvic floor muscle strength (PFMS), posterior bladder angle (PBA) measured by transperineal ultrasound, bladder neck descent (BND) measured by transperineal ultrasound, postpartum SUI, or POP. (4) Study designs considered eligible were observational studies, including cohort studies, case-control studies, and cross-sectional studies. Accordingly, when labor status was unavailable, the exposure was classified as reported elective CSD rather than confirmed prelabor elective CSD.

Studies were excluded if they: (1) did not provide a direct comparison between elective CSD and VD, (2) did not report extractable data for the outcomes of interest, (3) were reviews, conference abstracts without full text, case reports, editorials, or animal studies, or (4) included mixed cesarean delivery indications without clearly distinguishing elective CSD from emergency or intrapartum cesarean delivery when separate data were unavailable.

### Information sources and search strategy

We systematically searched MEDLINE via PubMed, Embase, Web of Science Core Collection, and the Cochrane Library from database inception to 31 March 2026. The strategy combined four concepts: cesarean delivery, vaginal delivery, transperineal/perineal ultrasound, and postpartum pelvic floor outcomes. Database-specific search builders, controlled vocabulary and field tags are reported in Supplementary Table S1. The archived reference-library export confirmed a combined pre-deduplication yield of 239 records but did not retain reliable source-database tags for every record; therefore, database-specific retrieval counts were not reconstructed retrospectively. Reference lists of included studies and relevant reviews were also screened manually.

### Study selection

All records retrieved were imported into a reference management system and duplicates were removed. Two reviewers independently screened titles and abstracts for potential eligibility. Full texts of potentially relevant records were obtained and assessed against the inclusion criteria. Disagreements were resolved through discussion; when consensus could not be reached, a third reviewer adjudicated. The study selection process was summarized using a PRISMA flow diagram.

### Data extraction

Two reviewers independently extracted data using a standardized form. Extracted items included: first author and publication year, country, study design, sample size (CSD and VD), maternal age, maternal BMI, postpartum assessment time, PFMS measurement method where available, ultrasound parameters and measurement conditions, reporting of elective CSD definition and labor/emergency cesarean exposure, and reported outcomes (PFMS, PBA, BND, SUI, POP). Each study was assigned to the outcome-specific synthesis only when extractable data for that outcome were available. Where key information was missing or unclear, it was recorded as not reported.

### Risk of bias and methodological quality assessment

Risk of bias was assessed using the Risk Of Bias In Non-randomized Studies of Interventions (ROBINS-I) tool for the effect of assignment to reported delivery mode (17). Prespecified confounders included maternal age, parity, body mass index, obstetric indication, labor exposure before cesarean delivery, instrumental delivery or perineal trauma, fetal birth weight, baseline pelvic floor status, and postpartum assessment time. The seven ROBINS-I domains were confounding, selection of participants, classification of interventions, deviations from intended interventions, missing data, measurement of outcomes, and selection of the reported result. Domain-level and overall judgments were categorized as low, moderate, serious, critical, or no information. Assessments were made at the result level; when a study contributed more than one outcome, Supplementary Table S2 reports the most conservative domain judgment across the results included in this review. The Newcastle-Ottawa Scale (NOS) and Agency for Healthcare Research and Quality (AHRQ) checklist were retained as complementary methodological-quality summaries rather than substitutes for ROBINS-I. Two reviewers assessed studies independently and resolved disagreements by consensus. Detailed ROBINS-I judgments are provided in Supplementary Table S2.

### Data synthesis and statistical analysis

When there was evidence from at least two studies reporting a similar outcome, meta-analysis was performed. For PFMS, standardized mean difference (SMD) was used because the included studies assessed pelvic floor muscle strength using different methods and potentially different scales. For PBA, mean difference (MD) was used because all included studies reported the angle in degrees. BND was not pooled because only two studies were available and measurement definitions differed. For dichotomous outcomes (SUI and POP), pooled effect estimates were calculated using risk ratios (RRs). Heterogeneity was assessed using Cochran's *Q*-test and the *I*^2^ statistic. Based on expected clinical and methodological heterogeneity, including differences in postpartum assessment time, ultrasound techniques, outcome definitions, and study design, a random-effects model was used as the primary model.

### Sensitivity and additional analyses

Sensitivity analyses were performed by sequentially removing one study at a time to evaluate the stability of pooled results. Because postpartum assessment times varied substantially, we examined whether formal time-based subgroup analyses were feasible using prespecified categories of early postpartum assessment (approximately 8 weeks or earlier), later follow-up, and not reported/unclear timing. Formal subgroup analysis was not performed when fewer than two studies were available within a clinically meaningful stratum; in those situations, the influence of assessment timing was addressed narratively in the interpretation of each outcome. Publication bias was evaluated using funnel plots and statistical tests (e.g., Egger's test) only when at least 10 studies were available for a given outcome.

### Certainty of evidence

Certainty of evidence for each outcome was evaluated using the GRADE framework (18). Because ROBINS-I was used, each body of evidence was initially considered high certainty and was then rated down for risk of bias, inconsistency, indirectness, imprecision, and publication bias. A serious overall ROBINS-I judgment was treated as a two-level downgrade for risk of bias. Factors that could increase certainty, including a large magnitude of association, dose-response gradient, and plausible residual confounding, were also considered. Certainty was categorized as high, moderate, low, or very low. The GRADE summary of findings and explicit reasons for rating are presented in Supplementary Table S3.

## Results

### Study selection

**Figure 1:**
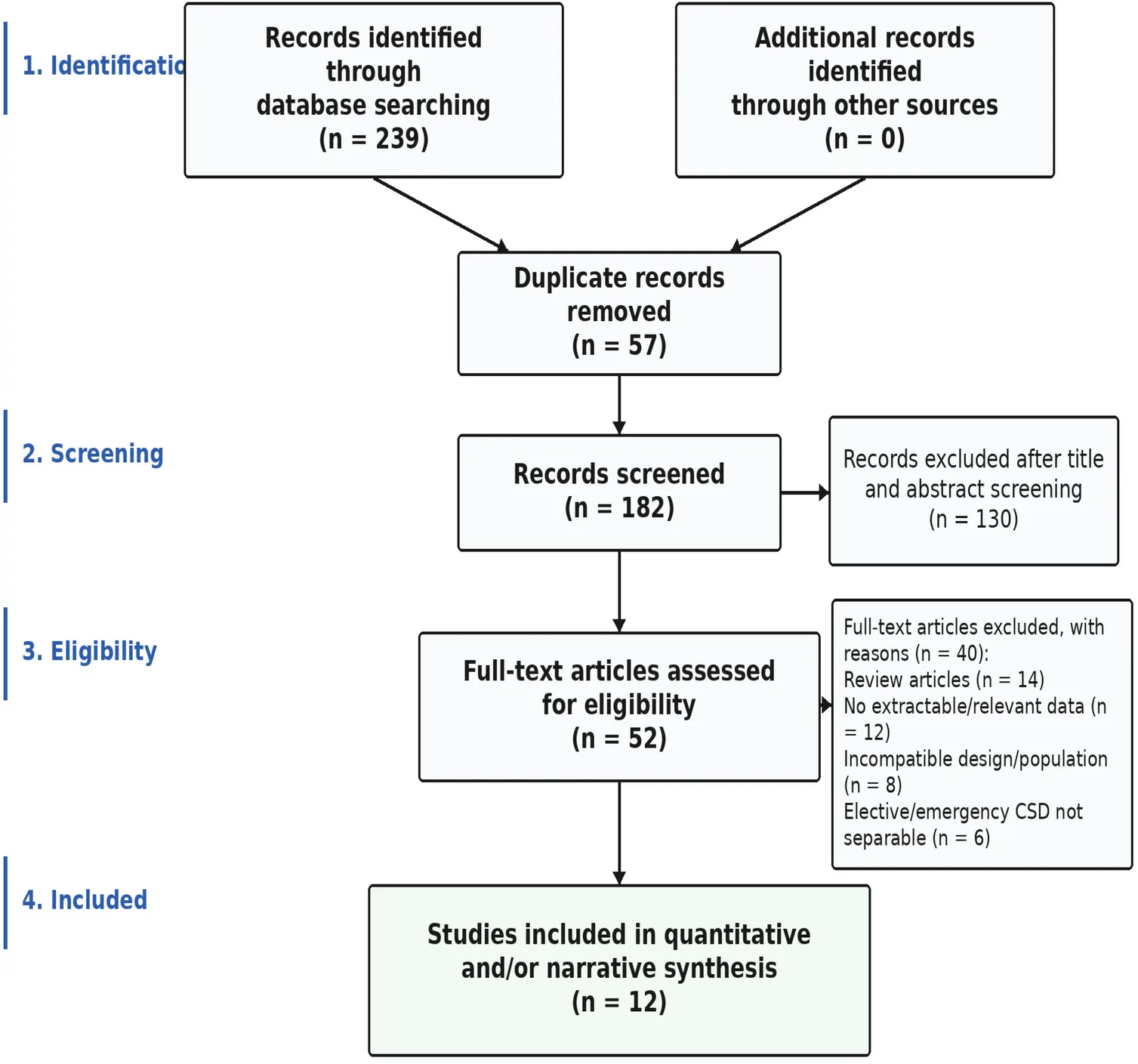
PRISMA flow diagram of study selection and inclusion in outcome-specific quantitative and/or narrative synthesis.

The literature search identified a verified aggregate of 239 records across the four databases, with no additional records retrieved from other sources. After removing 57 duplicates, 182 records remained for title and abstract screening, of which 130 were excluded as irrelevant. A total of 52 full-text articles were assessed for eligibility. Forty full-text articles were excluded with the following reasons: review articles (*n* = 14), no extractable or relevant outcome data (*n* = 12), incompatible study design or population (*n* = 8), and inability to distinguish elective from emergency cesarean delivery when separate data were unavailable (*n* = 6). Ultimately, 12 studies were included in the systematic review and in at least one outcome-specific quantitative or narrative synthesis. The selection process is summarized in the PRISMA flow diagram (Figure 1).

### Characteristics of included studies

A total of 12 studies were included in the systematic review and in at least one outcome-specific quantitative or narrative synthesis (Table 1), comprising 4 cohort studies (19–22), 1 case-control study (23), and 7 cross-sectional studies (3, 24–29). Studies were conducted in Brazil (*n* = 3), China (*n* = 4), Iran (*n* = 2), Norway (*n* = 1), Italy (*n* = 1), and Switzerland (*n* = 1). Sample sizes varied widely, ranging from CSD 29 vs. VD 193 in Hilde 2013 to CSD 928 vs. VD 1274 in Zhang 2024. Postpartum assessment times varied substantially: early postpartum evaluations were performed at 42–60 days (19), approximately 6 weeks (20, 27), 63.34 ± 16.96 days (23) and 6–8 weeks (22); one cohort study assessed outcomes at 1–3 years postpartum (21); and several cross-sectional studies did not report a specific postpartum time point (3, 24, 26, 28, 29). Table 1 includes a dedicated column reporting how elective CSD and labor/emergency cesarean exposure were described in each included study. For outcome-specific analyses, PFMS was extracted from five studies (19–21, 24, 25), PBA from three studies (22, 23, 27), BND from two studies (22, 23), SUI from six studies (3, 25–29), and POP from four studies (25–27, 29). Because postpartum timing was unevenly distributed and often not reported, the pooled estimates were interpreted as broad postpartum associations rather than time-specific effects. Labor exposure before cesarean delivery was not explicitly reported in most included studies; therefore, cesarean groups could not uniformly be confirmed as true prelabor elective procedures.

**Table 1:** Characteristics of included studies.

**Study**	**Country**	**Study design**	**Sample size**	**Age (years)**	**Maternal BMI (kg/m²)**	**Postpartum assessment time**	**Outcomes in present synthesis**	**Reported elective CSD/pre-CSD labor**
Caroci (19)	Brazil	Cohort study	CSD: 37	21.4 ± 5.1	NR	42–60 days	PFMS	Elective CSD extractable; pre-CSD labor NR
			VD: 73					
Batista (25)	Brazil	Cross-sectional study	CSD: 30	CSD: 30.5 ± 5.4	CSD: 24.3 ± 4.4	NR	PFMS, SUI, POP	Elective CSD extractable; pre-CSD labor NR
			VD: 31	VD: 32.3 ± 5.8	VD: 24.1 ± 4.2			
Afshari (24)	Iran	Cross-sectional study	CSD: 86	CSD: 28.9 ± 5.6	CSD: 26.9 ± 5.1	NR	PFMS	Elective CSD extractable; pre-CSD labor NR
			VD: 73	VD: 28.7 ± 5.3	VD: 26.8 ± 5.3			
Hilde (20)	Norway	Cohort study	CSD: 29	28.7 ± 4.3	NR	6 weeks	PFMS	Elective CSD extractable; pre-CSD labor NR
			VD: 193					
Cosimato (27)	Italy	Cross-sectional study	CSD: 98	31 ± 5	22.68 ± 3	6 weeks	PBA, SUI, POP	Elective CSD extractable; pre-CSD labor NR
			VD: 102					
Hongliang (23)	China	Case–control study	CSD: 78	CSD: 29.98 ± 3.40	CSD: 27.05 ± 2.95	63.34 ± 16.96 days	PBA, BND	Elective CSD extractable; emergency/mixed CSD separable; pre-CSD labor NR
			VD: 78	VD: 29.61 ± 2.35	VD: 27.13 ± 3.00			
Baud (26)	Switzerland	Cross-sectional study	CSD: 208	CSD: 37.4 ± 5.4	NR	NR	SUI, POP	Elective CSD extractable; pre-CSD labor NR
			VD: 309	VD: 36.6 ± 5.3				
Zhang (29)	China	Cross-sectional study	CSD: 928	32.37 ± 3.8	NR	NR	SUI, POP	Elective CSD extractable; emergency/mixed CSD separable; pre-CSD labor NR
			VD: 1274					
Sharifi (21)	Iran	Cohort study	CSD: 104	CSD: 29.1 ± 3.5	NR	1–3 years	PFMS	Elective CSD extractable; pre-CSD labor NR
			VD: 104	VD: 29.6 ± 3.7				
Wang (22)	China	Cohort study	CSD: 40	CSD: 23.62 ± 1.51	NR	6–8 weeks	PBA, BND, SUI	Elective CSD extractable; pre-CSD labor NR
			VD: 40	VD: 24.25 ± 1.77				
Grinbaum (28)	Brazil	Cross-sectional study	CSD: 46	CSD: 33.93 ± 7.19	CSD: 28.74 ± 4.91	NR	SUI	Elective CSD extractable; pre-CSD labor NR
			VD: 80	VD: 35.59 ± 8.21	VD: 26.77 ± 4.55			
Gao (3)	China	Cross-sectional study	CSD: 364	29.29 ± 3.75	NR	NR	SUI	Elective CSD extractable; pre-CSD labor NR
			VD: 481					

CSD, cesarean section delivery; VD, vaginal delivery; BMI, body mass index; PFMS, pelvic floor muscle strength; PBA, posterior bladder angle; BND, bladder neck descent; SUI, stress urinary incontinence; POP, pelvic organ prolapse; NR, not reported. Outcomes listed in Table 1 indicate outcomes included in the present quantitative or narrative synthesis. In the column ‘Reported elective CSD/pre-CSD labor,’ ‘elective CSD extractable' indicates that data labeled as elective CSD were separable in the original report; ‘emergency/mixed CSD separable' indicates that emergency or mixed cesarean data were excluded or separately available; and ‘pre-CSD labor NR' indicates that labor before cesarean delivery was not explicitly reported. Thus, reported elective CSD should not automatically be interpreted as confirmed prelabor cesarean delivery.

### Risk of bias and methodological quality assessment

The ROBINS-I assessment classified all 12 included studies as having a serious overall risk of bias, driven principally by residual confounding and selection into non-randomized delivery-mode groups. Incomplete reporting of labor exposure before cesarean delivery also created uncertainty in intervention classification. No study was judged to be at critical risk of bias. As complementary quality summaries, cohort-study NOS scores ranged from 7 to 9, the case-control study scored 8, and cross-sectional AHRQ scores ranged from 6 to 8 (Tables 2–4). Detailed domain-level ROBINS-I judgments are provided in Supplementary Table S2.

**Table 2:** Methodological quality scores of the Newcastle-Ottawa scale of cohort studies.

Study	Selection				Comparability of cohorts	Outcomes			Score
	Representativeness of cohort	Selection of nonexposed cohort	Ascertainment of exposure	Outcome lacking at the beginning		Outcome assessment	Sufficient follow-up time	Follow up adequacy	
Caroci (19)	★	★	★	☆	★☆	★	★	★	7
Hilde (20)	★	★	★	★	★☆	★	★	★	8
Sharifi (21)	★	★	★	★	★★	★	★	★	9
Wang (22)	★	★	★	★	★★	★	★	★	9

**Table 3:** Methodological quality scores of the Newcastle-Ottawa scale of case-control studies.

Study	Selection				Comparability	Exposure			Score
	Adequate definition of cases	Representativeness of the cases	Selection of controls	Definition of controls		Ascertainment of exposure	Same method of ascertainment for cases and controls	Non-Response rate	
Hongliang (23)	★	★	★	★	★☆	★	★	★	8

**Table 4:** Methodological quality scores of the agency for healthcare research and quality checklist for cross-sectional studies.

Study	1. Were the criteria for inclusion in the sample clearly defined?	2. Were the study subjects and the setting described in detail?	3. Was the exposure measured in a valid and reliable way?	4. Were objective, standard criteria used for measurement of the condition?	5. Were confounding factors identified?	6. Were strategies to deal with confounding factors stated?	7. Were the outcomes measured in a valid and reliable way?	8. Was appropriate statistical analysis used?	Score
Batista (25)	★	★	★	★	★	★	★	★	8
Afshari (24)	★	★	★	★	☆	☆	★	★	6
Cosimato (27)	★	★	★	★	★	★	★	★	8
Baud (26)	★	★	★	★	☆	★	★	★	7
Zhang (29)	★	★	★	★	★	★	★	★	8
Grinbaum (28)	★	★	★	★	★	★	★	★	8
Gao (3)	★	★	★	★	☆	☆	★	★	6

### Certainty of evidence

GRADE certainty was low for the SUI association and very low for PFMS, PBA, BND, and POP. For SUI, the body of evidence was downgraded two levels for serious risk of bias but not further for inconsistency or imprecision. Uncertainty about labor before cesarean delivery was handled within the ROBINS-I classification and confounding domains because the review question was explicitly framed as reported elective CSD vs. VD. The remaining outcomes were rated very low because serious risk of bias was accompanied by substantial inconsistency, imprecision, or the absence of a pooled estimate. Details are provided in Supplementary Table S3.

### Postpartum pelvic floor muscle strength

**Figure 2:**
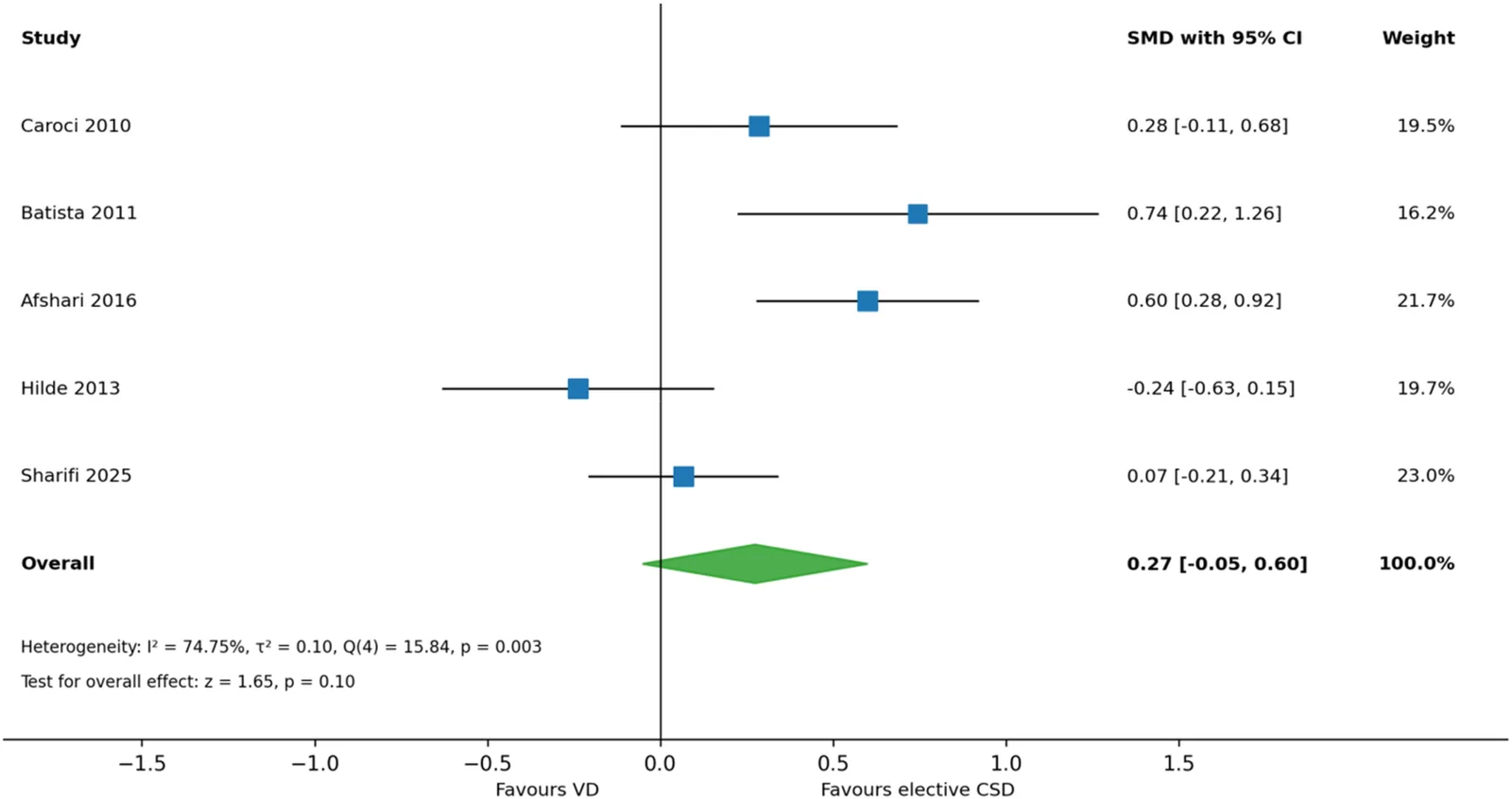
Forest plot comparing postpartum pelvic floor muscle strength after reported elective cesarean section delivery and vaginal delivery using standardized mean difference. Positive SMD values favor reported elective CSD; negative values favor VD.

Five studies reported postpartum PFMS and were included in the quantitative synthesis (19–21, 24, 25). Zhang 2024 was not included in this analysis because it did not report PFMS. Because the included studies used different PFMS assessment methods and scales, PFMS was pooled using SMD rather than MD. Using a random-effects DerSimonian-Laird model, reported elective CSD showed a non-significant trend toward higher PFMS compared with VD, with a pooled SMD of 0.27 (95% CI: −0.05 to 0.60; z = 1.65, *p* = 0.10) (Figure 2). Substantial heterogeneity was observed across studies (*I*^2^ = 74.75%, *τ*² = 0.10, Q(4) = 15.84, *p* = 0.003), suggesting meaningful between-study variability. Differences in PFMS measurement methods, postpartum timing, and participant characteristics likely contributed to this variability; accordingly, the pooled estimate remains uncertain and does not demonstrate superior PFMS after reported elective CSD.

### Posterior bladder angle

**Figure 3:**
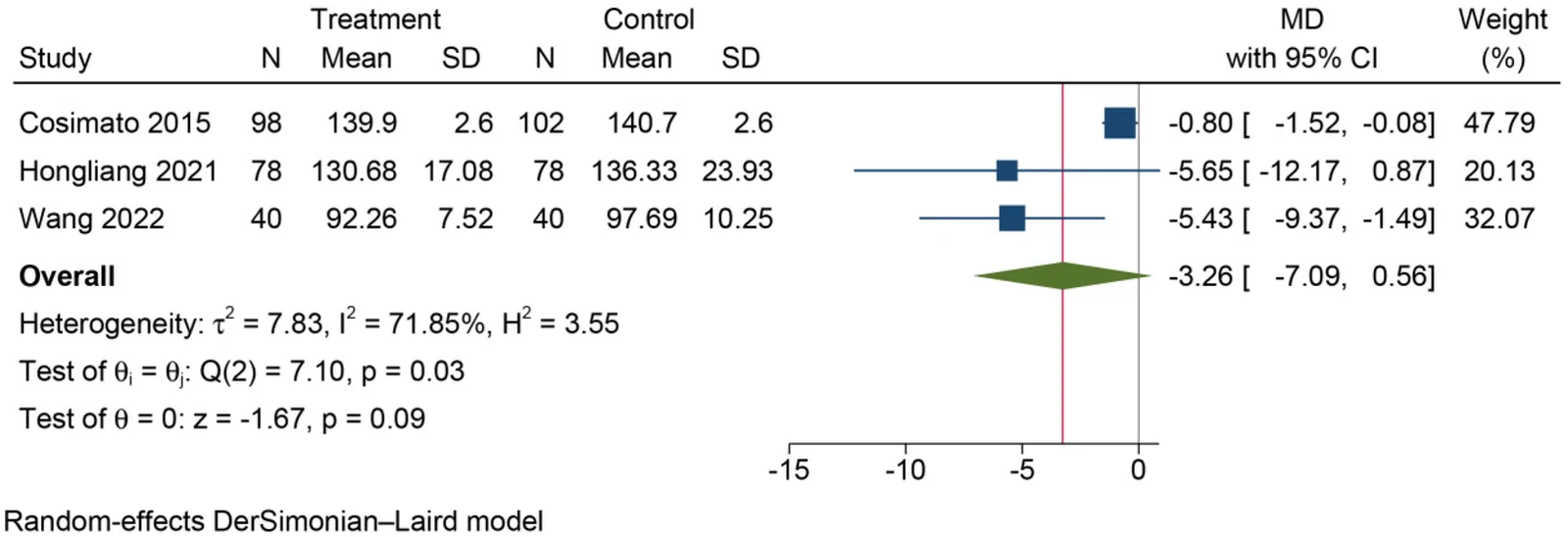
Forest plot comparing postpartum posterior bladder angle after reported elective cesarean section delivery and vaginal delivery. Negative MD values indicate a lower PBA in the reported elective CSD group; positive values indicate a higher PBA.

Three studies compared the PBA measured by transperineal ultrasound between reported elective CSD and VD in the postpartum period (22, 23, 27). Using a random-effects DerSimonian-Laird model, the pooled analysis showed no statistically significant difference in PBA between the two delivery modes, with an overall MD of −3.26 (95% CI: −7.09 to 0.56, *z* = −1.67, *p* = 0.09) (Figure 3). Substantial heterogeneity was observed (*I*^2^ = 71.85%, *τ*^2^ = 7.83, Q(2) = 7.10, *p* = 0.03), indicating notable between-study variability. While two studies suggested a smaller PBA after reported elective CSD (22, 27), the third study (23) showed an imprecise estimate crossing the null, which may have contributed to heterogeneity. Differences in postpartum timing and ultrasound protocol may also have influenced this result.

### Bladder neck descent

Two studies reported BND measured by transperineal ultrasound after childbirth (22, 23). Because only two studies were available and their measurement definitions and postpartum assessment windows differed, BND was summarized narratively rather than pooled. The limited evidence precludes a definitive comparison of bladder neck mobility between reported elective CSD and VD.

### Postpartum stress urinary incontinence

**Figure 4:**
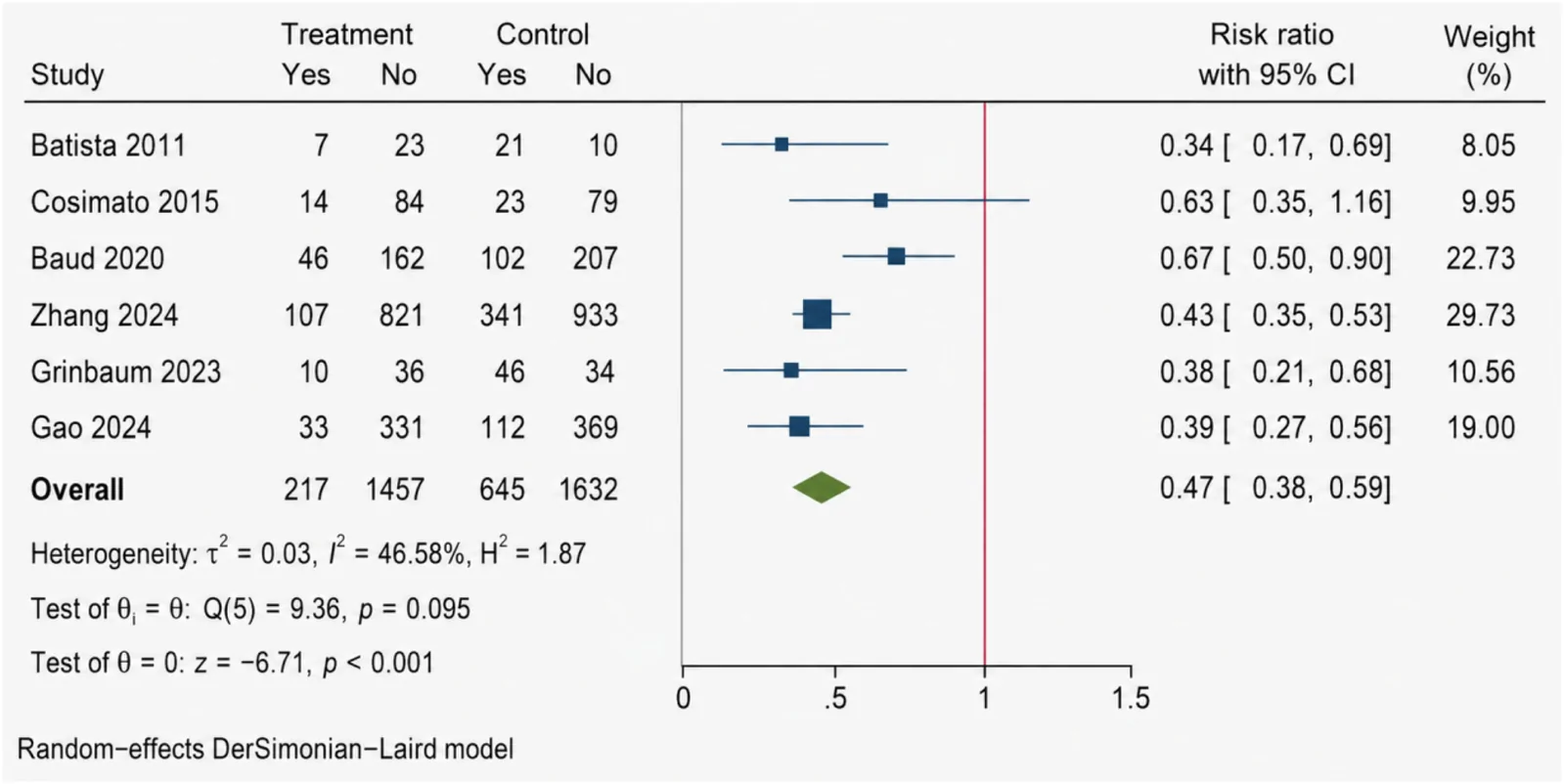
Forest plot comparing postpartum stress urinary incontinence after reported elective cesarean section delivery and vaginal delivery. RR < 1 favors reported elective CSD; RR > 1 favors VD.

Six studies reported postpartum SUI and were included in the meta-analysis (3, 25–29). Using a random-effects DerSimonian-Laird model, reported elective CSD was associated with a significantly lower risk of postpartum SUI compared with VD, with a pooled RR of 0.47 (95% CI: 0.38 to 0.59, *z* = −6.71, *p* < 0.001) (Figure 4). Moderate heterogeneity was observed across studies (*I*^2^ = 46.6%, *τ*^2^ = 0.03, Q(5) = 9.36, *p* = 0.095). The direction of the SUI estimate was generally consistent, although effect magnitude varied across study designs, assessment times, and outcome definitions. Because labor status was often unreported, this estimate pertains to groups described as elective CSD and not uniformly confirmed prelabor procedures.

### Postpartum pelvic organ prolapse

**Figure 5:**
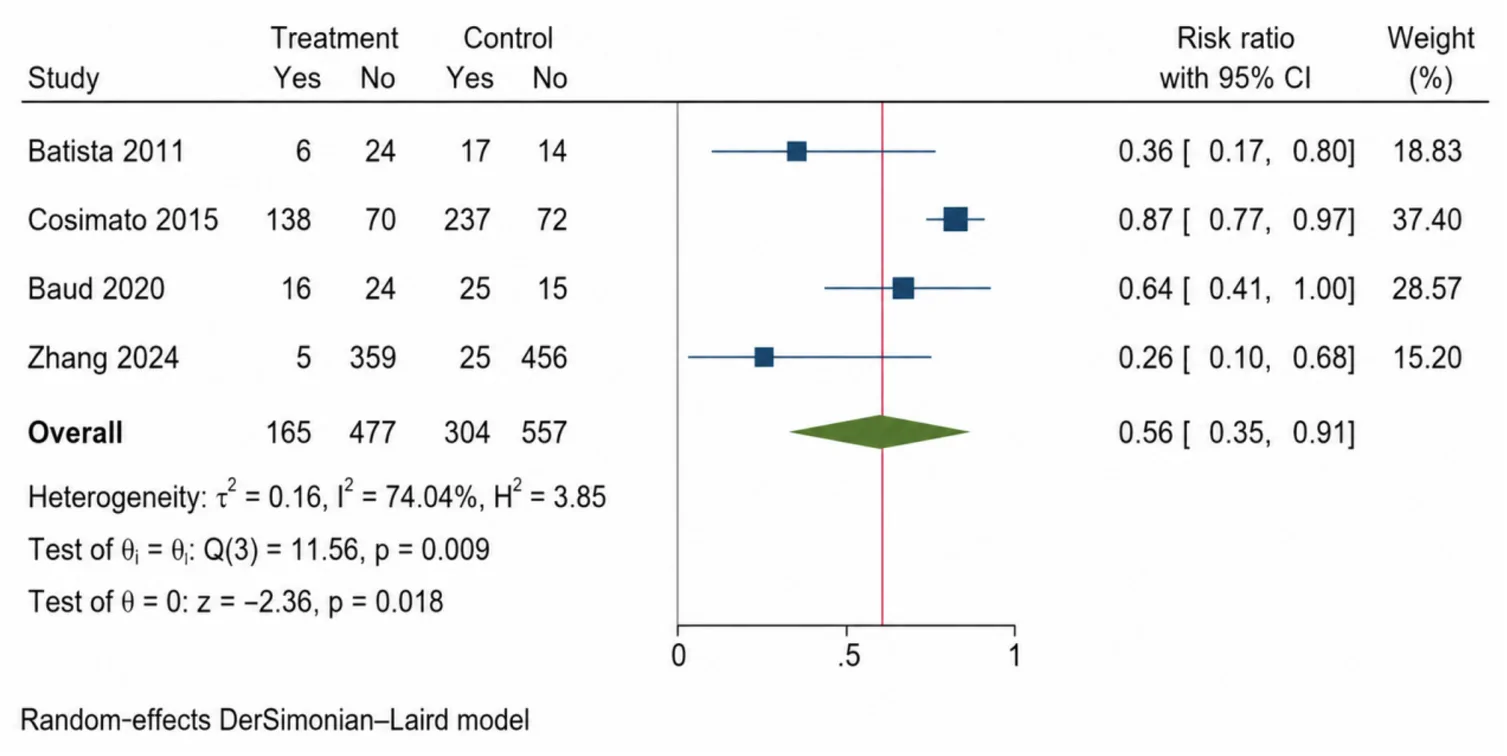
Forest plot comparing postpartum pelvic organ prolapse after reported elective cesarean section delivery and vaginal delivery. RR < 1 favors reported elective CSD; RR > 1 favors VD.

Four studies reported postpartum POP and were included in the meta-analysis (25–27, 29). Using a random-effects DerSimonian-Laird model, reported elective CSD was associated with a lower risk of POP compared with VD, with a pooled RR of 0.56 (95% CI: 0.35 to 0.91, z = −2.36, *p* = 0.018) (Figure 5). However, this estimate was based on only four observational studies and showed substantial heterogeneity (*I*^2^ = 74.0%, *τ*^2^ = 0.16, Q(3) = 11.56, *p* = 0.009); it is therefore less robust than the SUI finding.

## Discussion

This systematic review and meta-analysis synthesized transperineal ultrasound-based and clinically relevant outcomes comparing reported elective CSD with VD in postpartum women. Reported elective CSD was associated with a lower risk of postpartum SUI. The POP estimate also favored reported elective CSD, but was less robust because it was based on only four studies with substantial heterogeneity. PFMS showed a non-significant trend, PBA did not differ significantly, and BND was summarized narratively because only two studies with differing measurement definitions were available. Importantly, labor exposure before cesarean delivery was incompletely reported in most studies. The findings therefore represent associations with reported elective CSD and do not provide definitive evidence for uniformly confirmed prelabor cesarean delivery. Consistent with these limitations, GRADE certainty was low for SUI and very low for the remaining outcomes.

### Interpretation in the context of existing evidence

The PFMS finding showed a non-significant trend favoring reported elective CSD, which may be biologically plausible because VD-related stretching and microtrauma can affect pelvic floor neuromuscular performance during recovery. However, differences in PFMS measurement methods and postpartum assessment windows limited clinical interpretation and required the use of SMD rather than MD. Postpartum pelvic floor rehabilitation can improve pelvic floor muscle contraction and prolapse-related outcomes, underscoring that delivery mode does not fully determine recovery and that modifiable postnatal interventions remain relevant (14, 30).

### Interpretation of PBA and BND findings

An important finding was the mismatch between the clinical outcomes (SUI and POP) and some ultrasound-derived markers. The absence of a statistically significant difference in PBA may be related to differences in measurement technique, patient positioning, bladder volume, and Valsalva effort, all of which were not consistently standardized across studies. Reliability work and expert discussion also suggest that consistent imaging planes and measurement conditions are essential for comparability between cohorts (31). In addition, postpartum healing is dynamic: early edema and soft-tissue remodeling may distort pelvic dimensions and may mask or exaggerate between-group differences depending on assessment timing (32).

Only two studies reported BND, with differing measurement definitions and postpartum assessment windows. Accordingly, this outcome was summarized narratively, and the available evidence does not support a definitive comparison of bladder neck mobility between delivery groups.

The clinical significance of small ultrasonographic differences should also be interpreted cautiously. PBA and BND are sensitive to imaging plane, bladder volume, patient effort during Valsalva, and examiner technique. Therefore, small between-group differences in these markers should not be overinterpreted as evidence of clinically meaningful pelvic floor dysfunction without corresponding symptom or functional data.

### Clinical and translational implications

From a postnatal care perspective, these findings support the value of objective pelvic floor assessment within postpartum follow-up pathways. Transperineal ultrasound may help identify pelvic floor trauma and prolapse-related changes (33), and may be combined with clinical parameters or physiologic evaluations, such as pelvic electromyography, to improve risk stratification for postpartum SUI in real-world settings (15, 16). Such approaches are consistent with the broader goal of individualized pelvic floor care across the reproductive life course (1). No included study directly evaluated a rehabilitation intervention or compared rehabilitation programs. Accordingly, the present findings may inform risk identification and follow-up, but they do not establish a preferred rehabilitation protocol.

From a counseling perspective, the results should not be interpreted as evidence that elective CSD should be selected solely to prevent postpartum pelvic floor dysfunction. Delivery-mode decisions require shared decision-making that incorporates obstetric indication, maternal preference, fetal considerations, surgical risks, future reproductive plans, and access to postpartum rehabilitation. The present observational findings may inform pelvic floor risk discussions within this broader maternal-fetal framework but should not determine delivery mode (2). The SUI association was supported by six studies with moderate heterogeneity, whereas the POP estimate was based on four studies and was less robust.

Women undergoing reported elective CSD may differ systematically from women delivering vaginally in baseline pelvic floor risk, obstetric indication, maternal preference, fetal factors, and clinical management. Because these differences were rarely fully adjusted for, selection bias and residual confounding may have contributed to the observed SUI association and to the less robust POP estimate.

Heterogeneity across outcomes was moderate to substantial and may be explained by several factors: (1) variation in postpartum assessment timing, ranging from early postpartum evaluation to long-term follow-up or unreported timing; (2) variability in ultrasound protocol, operator technique, patient position, bladder volume, and Valsalva instruction; (3) different PFMS measurement methods and scales; (4) inconsistent reporting of whether elective CSD occurred before labor; and (5) heterogeneous adjustment for confounders. These sources of variability mean that the pooled estimates should not be interpreted as effects at a specific postpartum time point.

Future studies should prioritize well-designed prospective cohorts using standardized ultrasound protocols and prespecified postpartum time points. Reporting should include bladder volume, patient position, Valsalva coaching, PFMS measurement method, and whether cesarean delivery occurred before labor (31). Future research should also distinguish elective from emergency cesarean delivery and record labor exposure, because intrapartum processes may affect postpartum pelvic floor morphology (12). Imaging endpoints should be linked more clearly to patient-reported symptoms and functional outcomes, because ultrasound abnormalities do not always indicate symptomatic disease. Emerging automated ultrasound analysis may improve scalability and reduce operator dependence, supporting more consistent postpartum imaging evaluation (9).

### Strengths and limitations

This review has several limitations. First, all included studies were observational, and selection bias and residual confounding cannot be excluded. Second, PFMS measurement methods differed across studies; although SMD reduced scale-related incomparability, the pooled estimate remains clinically uncertain. Third, postpartum assessment times varied and were not consistently reported, preventing robust time-based subgroup analyses. Fourth, although elective CSD data were extractable, labor exposure before cesarean delivery was incompletely reported in most studies. Consequently, the cesarean groups cannot be assumed to represent uniformly confirmed prelabor elective procedures, and labor-related pelvic floor changes may have influenced comparisons with VD. Fifth, BND was reported in only two studies and was summarized narratively. In addition, the archived reference-library export did not preserve reliable source-database tags for every record; therefore, the aggregate pre-deduplication yield could be verified, but database-specific retrieval counts could not be reconstructed without estimation. Finally, outcome definitions and covariate adjustment varied across studies, which may have influenced the pooled estimates, particularly the heterogeneous POP result. The serious ROBINS-I judgments and the low-to-very-low GRADE certainty further indicate that the pooled associations should not be interpreted as causal effects.

Furthermore, our analysis was unable to incorporate critical parameters such as maximal levator hiatus area on Valsalva (LH max) and antero-posterior (AP) hiatal length. Although hiatal ballooning is a recognized independent risk factor for POP and SUI, the current comparative literature between elective CSD and VD lacks sufficient extractable data for these hiatal dimensions, highlighting a major gap that future prospective studies must address.

## Conclusion

Current observational evidence suggests that reported elective CSD may be associated with a lower risk of postpartum SUI compared with VD. The evidence for POP also favored reported elective CSD, but was less robust because it was based on only four studies with substantial heterogeneity. Although elective CSD data were extractable, labor exposure before cesarean delivery was incompletely reported in most studies; the findings should therefore be interpreted as associations with reported elective CSD, not as definitive evidence for true prelabor cesarean delivery. Evidence for PFMS and ultrasound-derived anatomical parameters remains uncertain because of limited study numbers, inconsistent measurement methods, and variable postpartum assessment timing. These findings may inform hypothesis generation and individualized counseling, but should not be used as the sole basis for delivery-mode decisions. GRADE certainty was low for SUI and very low for all other outcomes. The review did not evaluate rehabilitation interventions and therefore cannot support a specific rehabilitation plan.

## Data Availability

The original contributions presented in the study are included in the article/Supplementary Material, further inquiries can be directed to the corresponding author/s.
