# Supplementary material for: Transperineal ultrasound–based outcomes of postpartum pelvic floor recovery after elective cesarean section versus vaginal delivery: a systematic review and meta-analysis
Source: Front Glob Womens Health. 2026 Aug 12;7:1808222. doi: 10.3389/fgwh.2026.1808222 (PMC13506370; doi:10.3389/fgwh.2026.1808222)
Supplement: Supplementary file 1 [file Datasheet1.docx]

Supplementary Material

**Transperineal Ultrasound–Based Outcomes of Postpartum Pelvic Floor Recovery After Elective Cesarean Section Versus Vaginal Delivery: A Systematic Review and Meta-Analysis**

# Supplementary Table S1. Database-specific search strategies

Search date: 31 March 2026. Coverage: database inception to the search date. No language or publication-status filter was applied in the electronic search.

## MEDLINE via PubMed

**Records retrieved:** Source-specific count not retained in archived export.

(("Cesarean Section"[Mesh] OR "cesarean section"[tiab] OR "caesarean section"[tiab] OR "cesarean delivery"[tiab] OR "caesarean delivery"[tiab] OR "elective cesarean"[tiab] OR "elective caesarean"[tiab]) AND ("Delivery, Obstetric"[Mesh] OR "vaginal delivery"[tiab] OR "vaginal birth"[tiab]) AND ("transperineal ultrasound"[tiab] OR "perineal ultrasound"[tiab] OR "pelvic floor ultrasound"[tiab] OR "four-dimensional ultrasound"[tiab] OR "4D ultrasound"[tiab]) AND ("Pelvic Floor"[Mesh] OR "pelvic floor"[tiab] OR "pelvic floor dysfunction"[tiab] OR "pelvic floor muscle strength"[tiab] OR "posterior bladder angle"[tiab] OR "bladder neck descent"[tiab] OR "stress urinary incontinence"[tiab] OR "pelvic organ prolapse"[tiab] OR "levator hiatus"[tiab]))

## Embase

**Records retrieved:** Source-specific count not retained in archived export.

('cesarean section'/exp OR 'cesarean section':ti,ab,kw OR 'caesarean section':ti,ab,kw OR 'cesarean delivery':ti,ab,kw OR 'caesarean delivery':ti,ab,kw OR 'elective cesarean':ti,ab,kw OR 'elective caesarean':ti,ab,kw) AND ('vaginal delivery'/exp OR 'vaginal delivery':ti,ab,kw OR 'vaginal birth':ti,ab,kw) AND ('transperineal ultrasound':ti,ab,kw OR 'perineal ultrasound':ti,ab,kw OR 'pelvic floor ultrasound':ti,ab,kw OR 'four dimensional ultrasound':ti,ab,kw OR '4d ultrasound':ti,ab,kw) AND ('pelvic floor'/exp OR 'pelvic floor':ti,ab,kw OR 'pelvic floor dysfunction':ti,ab,kw OR 'pelvic floor muscle strength':ti,ab,kw OR 'posterior bladder angle':ti,ab,kw OR 'bladder neck descent':ti,ab,kw OR 'stress urinary incontinence':ti,ab,kw OR 'pelvic organ prolapse':ti,ab,kw OR 'levator hiatus':ti,ab,kw)

## Web of Science Core Collection

**Records retrieved:** Source-specific count not retained in archived export.

TS=(("cesarean section" OR "caesarean section" OR "cesarean delivery" OR "caesarean delivery" OR "elective cesarean" OR "elective caesarean") AND ("vaginal delivery" OR "vaginal birth") AND ("transperineal ultrasound" OR "perineal ultrasound" OR "pelvic floor ultrasound" OR "four-dimensional ultrasound" OR "4D ultrasound") AND ("pelvic floor" OR "pelvic floor dysfunction" OR "pelvic floor muscle strength" OR "posterior bladder angle" OR "bladder neck descent" OR "stress urinary incontinence" OR "pelvic organ prolapse" OR "levator hiatus"))

## Cochrane Library

**Records retrieved:** Source-specific count not retained in archived export.

([mh "Cesarean Section"] OR "cesarean section":ti,ab,kw OR "caesarean section":ti,ab,kw OR "cesarean delivery":ti,ab,kw OR "caesarean delivery":ti,ab,kw OR "elective cesarean":ti,ab,kw OR "elective caesarean":ti,ab,kw) AND ([mh "Delivery, Obstetric"] OR "vaginal delivery":ti,ab,kw OR "vaginal birth":ti,ab,kw) AND ("transperineal ultrasound":ti,ab,kw OR "perineal ultrasound":ti,ab,kw OR "pelvic floor ultrasound":ti,ab,kw OR "four-dimensional ultrasound":ti,ab,kw OR "4D ultrasound":ti,ab,kw) AND ([mh "Pelvic Floor"] OR "pelvic floor":ti,ab,kw OR "pelvic floor dysfunction":ti,ab,kw OR "pelvic floor muscle strength":ti,ab,kw OR "posterior bladder angle":ti,ab,kw OR "bladder neck descent":ti,ab,kw OR "stress urinary incontinence":ti,ab,kw OR "pelvic organ prolapse":ti,ab,kw OR "levator hiatus":ti,ab,kw)

**Traceability note:** The archived reference-library export verified a combined pre-deduplication total of 239 records across the four databases. Reliable source-database tags were not retained for every citation; consequently, source-specific yields were not retrospectively estimated. The limitation is reported transparently in the manuscript and response letter.

# Supplementary Table S2. ROBINS‑I domain-level risk-of-bias assessment

Target effect: effect of assignment to reported delivery mode. Prespecified confounders included maternal age, parity, body mass index, obstetric indication, pre-cesarean labor exposure, instrumental delivery/perineal trauma, fetal birth weight, baseline pelvic floor status, and postpartum assessment time.

| **Study** | **Confounding** | **Selection** | **Classification** | **Deviations** | **Missing data** | **Outcome measurement** | **Reported result** | **Overall** |
| --- | --- | --- | --- | --- | --- | --- | --- | --- |
| Caroci 2010 | Serious | Moderate | Moderate | Low | Moderate | Moderate | Moderate | Serious |
| Batista 2011 | Serious | Serious | Moderate | Low | Moderate | Serious | Moderate | Serious |
| Afshari 2016 | Serious | Serious | Moderate | Low | Moderate | Moderate | Moderate | Serious |
| Hilde 2013 | Serious | Moderate | Moderate | Low | Low | Low | Moderate | Serious |
| Cosimato 2015 | Serious | Serious | Moderate | Low | Moderate | Moderate | Moderate | Serious |
| Hongliang 2021 | Serious | Serious | Moderate | Low | Moderate | Moderate | Moderate | Serious |
| Baud 2020 | Serious | Serious | Moderate | Low | Serious | Moderate | Moderate | Serious |
| Zhang 2024 | Serious | Serious | Moderate | Low | Moderate | Moderate | Moderate | Serious |
| Sharifi 2025 | Serious | Serious | Moderate | Low | Moderate | Moderate | Moderate | Serious |
| Wang 2022 | Serious | Moderate | Moderate | Low | Moderate | Moderate | Moderate | Serious |
| Grinbaum 2023 | Serious | Serious | Moderate | Low | Moderate | Moderate | Moderate | Serious |
| Gao 2024 | Serious | Serious | Moderate | Low | Moderate | Moderate | Moderate | Serious |

**Judgment key:** Low, Moderate, Serious, Critical, or No information. Overall judgments reflect the highest domain-level concern. Assessments were made at the result level; where a study contributed to multiple outcomes, the table reports the most conservative judgment across the results included in this review. Judgments were based on information reported in the study publications.

# Supplementary Table S3. GRADE summary of findings

| **Outcome** | **Evidence and effect estimate** | **Absolute effect** | **Certainty** | **Main reasons for rating** |
| --- | --- | --- | --- | --- |
| PFMS | 5 observational studies; SMD 0.27 (95% CI -0.05 to 0.60) | Not applicable | **Very low** | Serious ROBINS-I risk of bias (two-level downgrade); serious inconsistency (I² = 74.75%); serious imprecision; indirectness not serious; publication bias not assessable (<10 studies). |
| PBA | 3 observational studies; MD -3.26° (95% CI -7.09 to 0.56) | Not applicable | **Very low** | Serious ROBINS-I risk of bias (two-level downgrade); serious inconsistency (I² = 71.85%); serious imprecision; indirectness not serious; publication bias not assessable (<10 studies). |
| BND | 2 observational studies; narrative synthesis only | Not applicable | **Very low** | Serious ROBINS-I risk of bias (two-level downgrade); serious inconsistency in definitions/timing; very serious imprecision because no pooled estimate was possible; publication bias not assessable. |
| SUI | 6 observational studies; RR 0.47 (95% CI 0.38 to 0.59) | Control: 283/1000; reported elective CSD: 133/1000 (108–167); 150 fewer/1000 (116–176 fewer) | **Low** | Serious ROBINS-I risk of bias (two-level downgrade); inconsistency, indirectness, and imprecision not serious; publication bias not assessable (<10 studies). |
| POP | 4 observational studies; RR 0.56 (95% CI 0.35 to 0.91) | Control: 353/1000; reported elective CSD: 198/1000 (124–321); 155 fewer/1000 (32–230 fewer) | **Very low** | Serious ROBINS-I risk of bias (two-level downgrade); serious inconsistency (I² = 74.0%); serious imprecision due to few studies and wide CI; publication bias not assessable. |

**GRADE approach:** Because ROBINS‑I was used, each body of evidence started at high certainty. A serious overall ROBINS‑I judgment was treated as a two-level downgrade for risk of bias. Additional downgrades were applied for inconsistency and/or imprecision where indicated. *The review question concerns groups reported as elective CSD; uncertainty about pre-cesarean labor was therefore addressed within ROBINS‑I classification/confounding rather than as indirectness. No outcome included at least 10 studies, so formal publication-bias testing was not performed.

**Interpretation:** The SUI association has low-certainty evidence. Evidence for PFMS, PBA, BND, and POP is very uncertain. These findings should not be used as the sole basis for delivery-mode decisions or to prescribe a specific rehabilitation program.
